# Real-imaging cDNA-AFLP transcript profiling of pancreatic cancer patients: Egr-1 as a potential key regulator of muscle cachexia

**DOI:** 10.1186/1471-2407-12-265

**Published:** 2012-06-21

**Authors:** Alexander Skorokhod, Jeannine Bachmann, Nathalia A Giese, Marc E Martignoni, Holger Krakowski-Roosen

**Affiliations:** 1 Division of Preventive Oncology (G110), National Center for Tumor Diseases (NCT) Heidelberg, German Cancer Research Center (DKFZ), Im Neuenheimer Feld 581, 69120, Heidelberg, Germany; 2 Department of Surgery, Klinikum rechts der Isar, Technische Universität München, Ismaninger Str. 22, 81675, Munich, Germany; 3 Department of General Surgery, University of Heidelberg, ImNeuenheimer Feld, 110 69120, Heidelberg, Germany; 4 Institute of Molecular Biology and Genetics, Ukrainian Academy of Sciences, Zabolotnogo str. 150, 03143, Kiev, Ukraine

## Abstract

**Background:**

Cancer cachexia is a progressive wasting syndrome and the most prevalent characteristic of cancer in patients with advanced pancreatic adenocarcinoma. We hypothesize that genes expressed in wasted skeletal muscle of pancreatic cancer patients may determine the initiation and severity of cachexia syndrome.

**Experimental design:**

We studied gene expression in skeletal muscle biopsies from pancreatic cancer patients with and without cachexia utilizing Real-Imaging cDNA-AFLP-based transcript profiling for genome-wide expression analysis.

**Results:**

Our approach yielded 183 cachexia-associated genes. Ontology analysis revealed characteristic changes for a number of genes involved in muscle contraction, actin cytoskeleton rearrangement, protein degradation, tissue hypoxia, immediate early response and acute-phase response.

**Conclusions:**

We demonstrate that Real-Imaging cDNA-AFLP analysis is a robust method for high-throughput gene expression studies of cancer cachexia syndrome in patients with pancreatic cancer. According to quantitative RT-PCR validation, the expression levels of genes encoding the acute-phase proteins α-antitrypsin and fibrinogen α and the immediate early response genes Egr-1 and IER-5 were significantly elevated in the skeletal muscle of wasted patients. By immunohistochemical and Western immunoblotting analysis it was shown, that Egr-1 expression is significantly increased in patients with cachexia and cancer. This provides new evidence that chronic activation of systemic inflammatory response might be a common and unifying factor of muscle cachexia.

## Background

Pancreatic cancer remains one of the most deadly tumor types, with the highest death-to-incidence ratio of all cancers. With a 5-year survival rate of less than 5% and a death-to-incidence ratio of 0.99, pancreatic ductal adenocarcinoma is currently one of the most aggressive gastrointestinal carcinomas [1]. The development of cachexia occurs in most patients with resectable and with unresectable pancreatic cancer, whose resting energy expenditure is generally increased. The term “cachexia” is used in the literature with different definitions and descriptions of clinical symptoms of a patient. It has been shown that a loss of weight exceeding 10% of the stable pre illness weight is correlated to a worse prognosis [2].

In the case of muscle wasting associated with cancer, the balance between protein synthesis and degradation is shifted markedly toward degradation, and there could be selective targeting of skeletal muscle gene products by different cachectic factors such as cytokines and tumor-derived compounds [3,4]. Weight loss of pancreatic cancer patients has been also related to the induction of acute-phase response, resulting in the loss of both fat and lean tissue. It has been demonstrated that a complex cascade of inflammatory responses may mediate alteration in metabolic processes, contributing to the development of cancer cachexia [5].

Unfortunately, patients with pancreatic cancer generally present in late stage of the disease, and frequently develop cachexia syndrome. This emphasizes the importance of early diagnosis, especially with the hope that intervention may be more effective in the early stages of disease. This also underscores the need for biomarkers, which may also contribute to the understanding of cancer cachexia syndrome [6]. Large-scale analysis of gene expression has been widely proposed as a powerful method for malignancy diagnosis, predicting invasion and metastasis through the identification of biomarkers. To our knowledge, however, transcriptome-wide analysis of muscle cachexia in patients has not yet been performed.

Here we present our study that focuses on the identification of common differentially expressed genes involved in muscle wasting progression. We hypothesized that gene expression patterns in the skeletal muscle of cancer patients will change under muscle wasting conditions, and that these changes may determine the initiation and severity of cachexia syndrome. We suggest that characterizing the skeletal muscle in response to cancer cachexia will provide new insights into gene regulation.

## Methods

### Patients

Patients with pancreatic cancer were selected for this study, as these patients usually experience severe weight loss (weight loss exceeding 10% of the stable body weight). All included patients gave written informed consent for tissue and data collection under the local ethics committee’s permission of the University of Heidelberg.

On admission to hospital, each patient was asked for his height and current weight. When a patient gave a history of weight loss, the corresponding time period was also documented. A patient was classified as cachectic when weight loss exceeded 10% of the stable pre-illness weight. It has been shown, that in patients with a history of weight loss exceeding 10% of the stable weight, the survival is significantly reduced [2,7].

*Histological diagnosis:* Two independent pathologists from the University of Heidelberg’s Department of Pathology confirmed the diagnosis according to the UICC guidelines [8].

### Muscle biopsies

Biopsy specimens were obtained from the rectus abdominis muscle of each patient during the initial phase of the operation. After skin incision and dissection through the subcutaneous fat, the anterior sheet of the rectus abdominis muscle was opened with scissors and a muscle biopsy specimen weighing about 50 mg was obtained and immediately frozen in liquid nitrogen.

### Real-Imaging cDNA-AFLP transcript profiling

mRNA was extracted from 20 mg rectus abdominis muscle using MagNA pure LC isolation system according to manufacturer’s instructions (Roche). After extraction, samples were immediately stored at −80°C until usage. mRNA quality was assessed by reverse transcription PCR (RT-PCR) with two housekeeping genes encoding ribosomal protein L13a (RPL13A) and β-actin. Negative controls for genomic contamination and reverse transcription inhibition were included.

The cDNA-AFLP procedure was performed as described previously with some modifications [9]. cDNA was synthesized from 100 ng mRNA according to the manufacturer’s instructions (cDNA Synthesis Kit, Roche). After restriction enzyme digestion, the cDNA fragments were ligated to oligomers introducing the priming sites for *Taq*I and *Mse*I. The cDNA pools were amplified with primers TaqI-Ø (5’-CTCGTAGACTGCGTACACGA-3’) and MseI-Ø (5’-GACGATGAGTCCTGAGTAA-3’).

cDNA-AFLP amplification was performed with 16 primer pairs MseI+2 and TaqI+2^IRD700^ (+2 states for additional two-nucleotide combination at 3’-end of primer) generating approximately 30,000 amplified fragments possessing 256 probe sets. IRD-labeled PCR products were resolved in 40 cm long 6% SequaGel XR gel (Biozym) using automated DNA sequencer DNAIR 4200 (Li-COR). After 1 h electrophoresis the gel was transferred onto the green grid paper and vacuum dried on 1.2-mm-thick Whatman paper. The dried gel was scanned at 700/800 nm (intensity 4.0, resolution 84 μm), obtaining binary data for differentially expressed tags (Odyssey Imaging System, Li-COR).

### Isolation and analysis of cDNA-AFLP tag fragments

The cDNA fragments of interest were excised from dried gel, followed by elution in 100 μl TE for 10 min at 95°C. 5 μl were re-amplified with TaqI-Ø and MseI-Ø primers. Specific fragments were verified by visualizing 5 μl PCR product on a 1.5% agarose gel. The amplified fragments were directly sequenced with the primers TaqI-Ø^IRD700^ and MseI-Ø^IRD800^ using the SequiTherm EXCEL II DNA sequencing kit (Epicentre) on the automated infrared sequencer DNAIR 4200 (Li-COR).

Sequencing data were aligned against BlastX and BlastN in the publicly available databases and clustered according to gene ontology using the nodes of the DNASpace software package (DNASIS MAX 2.6, Miraibio).

### Quantitative RT-PCR

Single-stranded cDNA was synthesized from 100 ng of mRNA according to the manufacturer’s instructions (RevertAid, Fermentas). Primers complementary to target genes were designed using Primer Express Software (Life Technologies) to amplify fragments in the range from 75 bp to 200 bp, close to the 3’-end of transcripts. A quantitative RT-PCR (qPCR) was performed with SYBR Green PCR Master Mix using GeneAmp 7300 (Life Technologies). qPCR reactions were performed two times in doublets, and the threshold cycle numbers were averaged. Levels of expression were normalized against RPL13A and β-actin.

### Immunohistochemistry

For immunohistochemistry, 5-μm sections of FFPE tissue were incubated in serum-free protein blocking buffer for 30 min, followed by overnight incubation at 4°C with 1:200 dilution of Egr-1 antibodies (588, Santa Cruz). A negative control for non-specific staining was performed with rabbit IgG. DAKO anti-rabbit polymer-HRPO was applied for signal visualization according to the manufacturer’s instructions (DAKO). Finally, sections were analyzed with an Olympus BX50 microscope using 40x-lens and software program “Cell^F 2.5” (Olympus).

### Protein extraction and immunoblotting

30 μg total protein lysate from the biopsy homogenate were electrophoresed on gradient 4-15% SDS-PAA gels (Bio-RAD). After undergoing electrophoresis, proteins were transferred semi-dried onto PVDF membrane using the Semi-Dry Blotter (Bio-RAD). After 1 h blocking, the membrane was exposed overnight at 4°C to primary anti-human rabbit Egr-1 antibody (588, Santa Cruz) and anti-human mouse β-tubulin antibody (3 F3-G2, Santa Cruz) at 1:200.

After washing the membrane was exposed to appropriate IRDye-700/800 labeled secondary antibodies: Alexa Fluor 680 goat anti-mouse for β-tubulin (Invitrogen) and IRDye-800 goat anti-rabbit for Egr-1 (Rockland) at 1:10,000. The amounts of Egr-1 and β-tubulin were determined and quantified by densitometric scanning of PVDF membranes at 700/800 nm (intensity 5.0, resolution 84 μm) using the ODYSSEY Infrared Imaging System (Li-COR). Individual band intensities were normalized against β-tubulin.

### Statistical analysis

All data are presented as means ± standard deviation. The significance of differences between groups (cachexia vs. no cachexia) was tested with Student’s *t*-test and Mann–Whitney *U* test. The statistical package SPSS for Windows (SPSS) was used for all statistical analyses. Differences were considered significant at the 0.05 level of confidence.

## Results

### Patient characteristics

We studied skeletal muscle biopsy samples of 23 patients diagnosed with histologically proven adenocarcinoma of the pancreas and treated at the Department of Surgery, University of Heidelberg, between November 2004 and April 2005. In ten of the patients weight loss exceeded 10% of the stable pre illness weight, while weight loss was absent or less than 10% of the stable weight in 13 patients. Clinical characteristics of these patients are summarized in Table 1 and Table 2. Tumor resection was performed in 17 patients (73.9%). Biopsies of the rectus abdominis muscle were obtained intraoperatively from pancreatic cancer patients with cachexia (n = 10) or without cachexia (n = 13). The resection rate was lower in patients with cachexia (50% vs. 92.3% P = 0.025). The median age was 65 years for cachectic patients and 66 years for non-cachectic patients, with lower and upper quartiles of 57 and 74 years for cachectic patients and 51 and 69 years for non-cachectic patients, with no significant difference between the examined groups. There was no significant difference in the performance status between patients with and without cachexia: every patient who was referred for operative treatment had a Karnofsky Index ≥70 (Table 1). In the examined patients there was no significant difference in CrP-levels in the serum – as a sign for systemic inflammation – preoperatively (P = 0.548). Furthermore, there was also no significant difference in protein levels between patients with and without cachexia (P = 0.19).

**Table 1:** Clinical characteristics of patients with pancreatic cancer with (N = 10) and without cachexia (N = 13): gender and performance status

**Parameter**		**No cachexia (N = 13) N [%]**	**Cachexia (N = 10) N [%]**
gender	male	9 [69.2]	5 [50]
	female	4 [30.8]	5 [50]
Karnofsky- Index	70	0 [0]	1 [10]
	80	1 [7.7]	2 [20]
	90	12 [92.3]	7 [70]

**Table 2:** Characteristics of patients with pancreatic cancer with (N = 10) and without cachexia (N = 13)

**Parameter**		**No cachexia (N = 13)**	**Cachexia (N = 10)**	**p-value**
age	median (lq/uq)	66 (51/69)	65 (57/74)	0,385
weight loss	median (lq/uq)	2,1 (0/5,5)	13,9 (10/19,2)	<0.001
CrP	median (lq/uq)	7.3 (5.7/11.8)	11.6 (3.1/25.6)	0.548
protein	median (lq/uq)	74.9 (70.8/77.9)	71.4 (66.4/75.6)	0.19
resectionrate		12 (92.3)	5 (50)	0.022
stage	UICC II	12 (92.3)	4 (40)	0.005
	UICC III	1 (6,7)	1 (10)	
	UICC IV	0	5 (50)	

**Figure 1:**
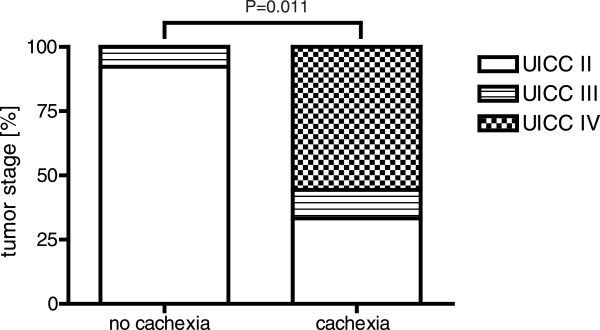
**UICC Classification of the 23 patients operated for pancreatic cancer.** Distribution of tumor stage of all pancreatic cancer patients was determined according to the 5th edition of the TNM classification of the Union International Contre Cancer (UICC). Median weight loss of the cachectic patients was 13.9% of initial body weight within 6 months comparing with the patients without cachexia in which the median weight loss was 2.1% respectively. The resection rate was lower in patients with cachexia (50% vs. 92.3%) UICC II: without distant metastases; UICC III: infiltration of celiac trunk and/or mesenteric artery; UICC IV: metastases in liver and/or peritoneal cavity according to guidelines UICC 2002) in patients either with (n = 10) or without cachexia (n = 13).

Initial staging of these patients was determined according to the 5th edition of the TNM classification of the International Union against Cancer. UICC classification of operated and examined patients (N = 23) is shown in Figure 1. Median weight loss of the cachectic patients was 13.9% of initial body weight within 6 months, compared to 2.1% in the patients without cachexia (P<0.001).

### Real-Imaging cDNA-AFLP screening

**Figure 2:**
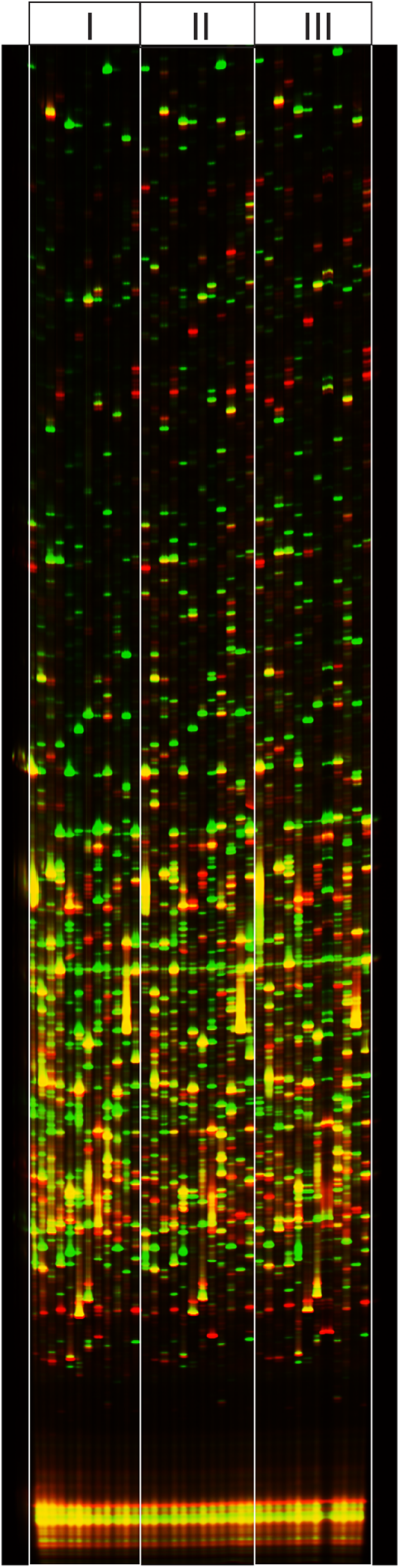
**cDNA-AFLP Transcript Profiling of pancreatic cancer patients.** cDNA-AFLP tag patterns amplified from mRNA of skeletal muscle biopsy samples of pancreatic cancer patients either with cachexia (IRD800-labeled, red bands) or without cachexia (IRD700-labeled, green bands) were loaded in the same lane according to their primer combinations and separated in a 8% denaturing polyacrylamide gel. Data were collected on automated Infrared Sequencer DNAIR 4200 (Li-COR Biosciences GmbH). Three different cDNA-AFLP tag profiles (i), (ii) and (iii) of wasted patients (red bands) were compared with one non-cachectic control (green bands).

We performed gene expression profile screening of the skeletal muscle of pancreatic cancer patients either with or without cachexia syndrome utilizing Real-Imaging cDNA-AFLP transcript profiling. Biopsy samples from 10 wasted patients and 13 patients without cachexia were compared by running the cDNA-AFLP reaction using a single primer combination. This comparison revealed that amplified tag patterns are almost identical within one group and differ between the groups (Additional file 1: Figure S1). Therefore, two biopsy samples from each group were further selected for preparative Real-Imaging cDNA-AFLP (Figure 2).

**Figure 3:**
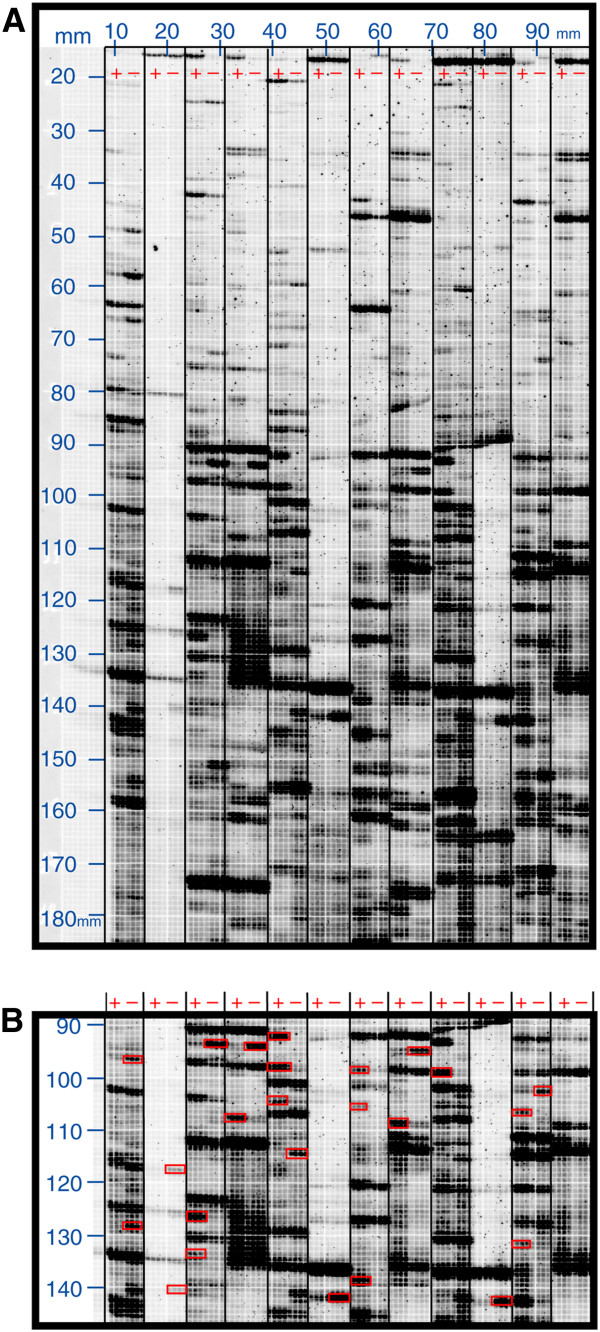
**Preparative Real-Imaging cDNA-AFLP Screening.A**) cDNA tag patterns were amplified with individual primer combinations Taq+2 and Mse+2 (Taq+2 was labeled with IRD700). After electrophoresis, the gel was transferred and dried on green grid paper and scanned using Odyssey Imaging System (Li-COR Biosciences GmbH). The lanes are occupied by cachexia-associated samples (+) and non-cachectic probes (−) allowing direct visual comparison. Grid coordinates are indicated in millimeters (mm). **B**) Differentially expressed tag patterns (marked in red boxes) were isolated for sequencing analysis.

Altered expression patterns were readily recognized by visual inspection and yielded approximately 400 cDNA fragments (Figure 3A). These cDNA fragments corresponding to differentially accumulating transcripts were excised from the dried preparative gels (Figure 3B). The accuracy of band excision was inspected by re-scanning. Sequence information was obtained by direct sequencing using primers MseI-Ø^IRD700^ and TaqI-Ø^IRD800^ on both strands.

### Analysis of differentially expressed genes

183 cDNA sequences were identified after a redundancy check of about 400 isolated tag fragments. They were clustered into groups according to their annotation fields. 127 cDNA fragments were referred to the gene transcripts that predominantly activated in cachectic patients, as well as 56 individual genes whose transcripts accumulated predominantly in the non-cachectic control group (Additional file 2: Table S1).

According to group classification, the identified genes provide molecular counterparts of muscle cachexia responsible for actin cytoskeleton rearrangement, actin filament disruption, extracellular matrix dysregulation, protein degradation, energy expenditure, tissue hypoxia, immediate early response, and acute-phase response. We identified a number of differentially expressed genes, which have already been characterized in relation to cancer cachexia or muscle atrophy. However, our results also indicate that there are other differentially expressed genes, which play unknown roles in muscle cachexia.

In wasted patients we observed the upregulation in gene expression of the contractive muscle proteins (Additional file 2: Table S1), correlating directly with other findings [10]. Individual differences were found in expression of the genes belonging to different proteolytic pathways (Additional file 2: Table S1, “Protein Degradation”). A number of genes encoding both transcription and translation factors as well as different ribosomal proteins were induced in response to muscle cachexia. In excellent agreement with previous findings, we observed the upregulation of class O type forkhead transcription factor 1 (FOXO1), which has been implicated as a key regulator of gene expression during skeletal muscle atrophy [11]. We have also identified alteration of the genes that were previously described in association with hypoxia (Additional file 2: Table S1, “Hypoxia”). Results of our cDNA-AFLP screening revealed that the genes encoding acute-phase response (APR) proteins fibrinogen α (FGA), fibrinogen γ (FGG), α_1_-antitrypsin, and α_2_-macroglobulin are induced in the skeletal muscle of pancreatic cancer patients with cachexia.

### Validation of cachexia-associated genes with qPCR

The differential gene expression level of 100 transcripts from cDNA-AFLP screening profile corresponding to the genes, which are associated with protein degradation, hypoxia, acute-phase response or with disorder in muscle tissue development, was validated by quantitative RT-PCR of the same skeletal biopsy samples (n = 23) using a Mann–Whitney *U* test. Candidate genes with a P-value threshold of 0.05 are presented in Table 3. qPCR analysis confirmed significant alteration of transcripts encoding α_1_-antitrypsin, α_2_-macroglobulin, fibrinogen α, immediate early-response genes Egr-1 and IER-5, pro-inflammatory cytokine IL-32, and hypoxia-related bHLH transcription factors Hes-1, DEC1 and DEC2. The gene expression level of fibrinogen γ (FGG) was not detectable by qPCR in our experiment. None of the above mentioned genes except APR proteins have been previously correlated with muscle cachexia of cancer patients. Aberrant overexpression (>60-fold) of Egr-1 gene was further elucidated.

**Table 3:** mRNA expression level of cDNA-AFLP candidate genes – cachexia vs. no cachexia

**Gene description**	**Fold change**	**Gene description**	**Fold change**
α_1_-antitrypsin (serpin)	18.61	fibrinogen α	6.65
α_2_-macroglobulin	4.77	forkhead transcription factor FOXO1A	5.22
ankyrin	0.41	immediate early response 5 (IER-5)	7.72
archvillin	22.04	karyopherin alpha (importin 5)	0.54
breast cancer-associated protein	9.47	mt translational release factor 1 (MTRF 1)	9,68
calsequestrin 1	22.03	myocyte enhancer factor 2 (MEF2)	13.77
cardiomyopathy associated protein 5	15.4	myosin regulatory light chain (MYLC)	5.63
cofilin 2	16.72	myosin heavy chain 7 (MYH7)	4.54
cyclin-dependent kinase inhibitor 1A (p21)	6.54	NK transcript (IL-32)	6.1
bHLH transcription factor DEC1	3.46	synaptopodin (SYNPO)	1.79
bHLH transcription factor DEC2	2.3	titin	4.19
bHLH transcription factor HES1	5.28	vimentin	2.39
early growth response 1 (Egr-1)	61.14	vinculin	2.68

### Egr-1 protein level is highly elevated in wasted skeletal muscle

**Figure 4:**
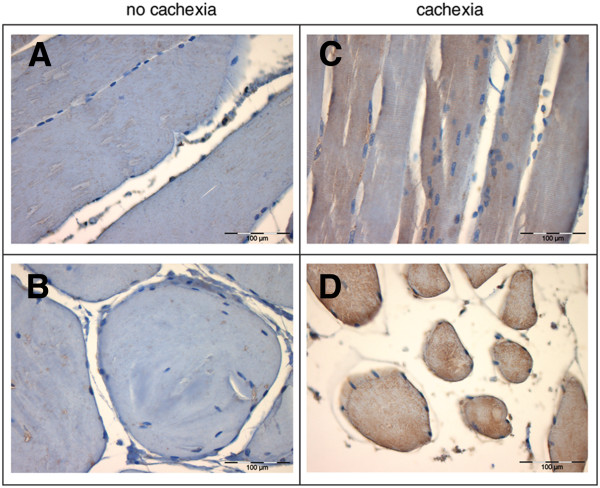
**Altered expression of Egr-1 in the skeletal muscle tissue of wasted pancreatic cancer patients.** Immunohistochemical staining of skeletal muscle tissue shows significantly enhanced expression of Egr-1 in wasted pancreatic cancer patients (**C**, **D**) compared with non-cachectic patients (**A**, **B**). **A**, **C** – longitudinal sections; **B**, **D** – transverse sections of skeletal muscle from pancreatic cancer patients. 40X original magnification.

To prove our cDNA-AFLP gene profiling strategy, followed by qPCR validation, the protein expression levels of transcription factor Egr-1 were further analyzed by immunostaining and Western immunoblotting. The expression sites of Egr-1 protein were identified both in longitudinal and transverse sections of the skeletal muscle from patients either with or without cachexia by immunohistochemical staining with Egr-1 antibodies. Comparison of tissue sections revealed a significantly stronger immunohistochemical signal of Egr-1 protein in the skeletal muscle tissue cells of patients with cachexia, as indicated by increased fluorescent staining (Figure 4). Similar results were obtained with fibrinogen α immunohistochemical staining (data not shown).

**Figure 5:**
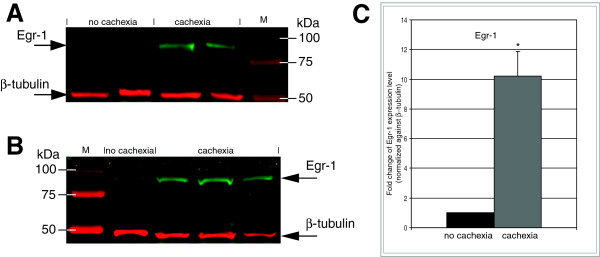
**Egr-1 protein is markedly induced in skeletal muscle of wasted pancreatic cancer patients.** Protein levels of skeletal muscle EGR-1 and β-tubulin (loading control) were determined by Western immunoblotting showing aberrant upregulation of Egr-1 in skeletal muscle of patients with cancer cachexia. Western immunoblotting of Egr-1 (green band, 82 kDa) and β-tubulin (red band, 55 kDa) compares (**A**) two non-cachectic with two cachexia-associated biopsy protein extracts and (**B**) one non-cachectic probe with three cachexia-associated skeletal muscle biopsy samples. Similar results were obtained in three independent experiments. M – coomassie-blue prestained protein marker. Full-length blots/gels are presented in Additional file 3: Figure S2. (**C**) Densitometric analysis of Egr-1 protein expression. Median rate = 10.23, P-value ≤ 0.005 according to Student’s *t*-test.

Furthermore, Egr-1 protein (82 kDa) was quantified in protein extracts from muscle biopsy samples by Western immunoblotting using densitometric quantitation of the appropriate bands against β-tubulin by infrared fluorescence detection directly on the PVDF membrane (Figure 5). Immunoblotting detection revealed significantly higher levels (median rate 10.3, P≤0.005, Student’s *t*-test) of Egr-1 expression in all wasted patients with pancreatic cancer compared with controls (Figure 5C). Concentrations of Egr-1 protein remained elevated among all wasted patients. In some non-cachectic controls the expression level of Egr-1 protein was underrepresented (Figure 5A).

Finally, immunohistochemistry and Western immunoblotting analysis confirmed the strong correlation of Egr-1 within the skeletal muscle of pancreatic cancer patients in response to cachexia.

## Discussion

Cachexia is present in around 80% of patients with cancer in advanced stages, inducing marked depletion of lean and fat mass, and in more than 20% of patients cachexia is responsible for death [12]. Whereas starvation and chronic malnutrition are associated with adaptive decreases in metabolic rate, cancer cachexia is characterized by altered metabolic processes and activation of catabolic pathways [13].

We hypothesized that alteration in gene expression within the skeletal muscle in response to advanced pancreatic cancer plays a crucial role in the development of the muscle cachexia syndrome. To test this hypothesis, we carried out the transcriptome-wide characterization of skeletal muscle biopsies utilizing the Real-Imaging cDNA-AFLP approach to identify those genes that are commonly induced in the skeletal muscle of pancreatic cancer patients either with or without muscle cachexia.

In contrast to other studies of skeletal muscle cancer cachexia based on cDNA microarray technique[14,15], our Real-Imaging cDNA-AFLP approach gives the ability to identify and annotate expressed genes without prior sequence knowledge making possible to distinguish among different transcripts from genes belonging to the same gene family or splice variants of the same gene. Importantly, our results indicate that molecular signatures of gene expression appear to be predictive of muscle cachexia, before we can measure this in the preoperative collected blood samples as there is no significant difference in protein levels or CrP-levels to detect. We suggest that in characterizing the skeletal muscle transcriptome, one might expect that different signal systems may work in combination to shift the disease stage of pancreatic cancer patients towards cachectic conditions.

We observed that transcript levels of contractile proteins in skeletal muscle of patients with cachexia change in the same direction. We observed more than 6-fold upregulation of cyclin-dependent kinase inhibitor p21 (Table 3). The mRNA levels of increased p21 mRNA expression in cachectic patients may reflect increased activity of myostatin, a negative regulator of muscle growth and development which is thought to induce cachexia [16]. The skeletal muscle mass appears to be significantly involved in the process of cancer cachexia in patients with pancreatic cancer, being the potential target of various tumor products which can activate protein degradation [17]. All these changes may indicate the abnormal metabolic processes shifting towards hypermetabolism and negative energy expenditure in patients with cachexia.

We have also observed the induction of genes encoding basic helix-loop-helix (bHLH) transcription factors HES1, DEC1, and DEC2, which are primarily associated with hypoxia (Table 3). As it was reported previously, hypoxia enhances Notch signaling and activates Notch-responsive promoters and increases expression of bHLH transcription factors [18]. HES1 and DEC1 (Table 3) are immediate downstream targets of Notch signaling inhibiting the transcriptional expression and/or function of lineage-specifying genes involved in myogenesis [19].

Our study demonstrates for the first time that the genes encoding acute-phase response (APR) proteins fibrinogen α, α_1_-antitrypsin and α_2_-macroglobulin are markedly upregulated not only in the liver but also in the skeletal muscle of wasted patients with pancreatic cancer (Table 3), which are associated with altered energy expenditure and increased protein metabolism of pancreatic cancer patients [20]. It has been shown that approximately 40 percent of pancreatic cancer patients show an acute-phase response at the time of diagnosis, and this amount increases to around 80 percent at the time of death [21].

While acute-phase response indicates systemic inflammation, the pro-inflammatory cytokines appear to mediate this response. Furthermore, it has been reported that non-immune tissues such as cardiac and skeletal muscle appears to express pro-inflammatory cytokines [22]. In excellent agreement with these facts, we observed 6-fold upregulation of interleukin IL-32 (Table 3), play an important role in inflammatory diseases as direct inducer of TNF-α [23].

Notably, in contrast to the previous study on human skeletal muscle cancer cachexia [14], we observed a more than 5-fold upregulation of FOXO1, a key regulator of gene expression during skeletal muscle atrophy [11] (p<0.05, Table 3). We suppose that it may be explained by more stringent selection of cachectic patients in our study with weigh loss criteria from 10 to 19% within 6 months (Table 1).

Importantly, together with an ongoing inflammatory response we observed the significant induction of early response genes Egr-1 and IER-5 in wasted pancreatic cancer patients (p<0.05, Table 3). Conceptually, Egr-1, the master switch regulating inflammatory parameters, can be viewed as a main initiator of inflammatory response, as it is directly involved in “early signal events”[24,25]. Egr-1 is also recognized as playing a critical role in the regulation of over 40 target genes, including tumor necrosis factor α (TNF-α), interleukin (IL)-1β, and IL-6, all of which can induce various features of cachexia syndrome in the skeletal muscle in animal models [26]. Together with elevated acute phase response gene expression, the Egr-1 may serve as a biomarker of muscle cachexia syndrome.

## Conclusions

This report provides the first evidence that Egr-1 is aberrantly upregulated under conditions of muscle wasting, underlining the general importance of pro-inflammatory response in cancer cachexia (Figures 4 and 5). Together with altered IER-5 and ongoing acute-phase response, it adds to the body of evidence that systemic inflammatory response contributes mainly to cancer cachexia progression mediating altered metabolic processes, considering this condition as a chronic systemic inflammatory disease.

In summary, taken together with previous works in the field, the current study supports a model of muscle wasting in which the major influence of cancer cachexia is based on the chronic inflammatory effects resulting in onset of acute-phase response. Furthermore, this report provides new insights into the inflammatory response involved in initiation and progression of cachexia by the activation of pro-inflammatory transcription factor Egr-1 and acute-phase response. How they are transduced to initiate the muscle cachexia remains to be investigated.

## Abbreviations

cDNA-AFLP, cDNA amplified fragment-length polymorphism; Egr-1, early growth response; 1IER-5: immediate early response 5; APR, Acute-phase response.

## Competing interests

All authors have completed the ICMJE uniform disclosure form at http://www.icmje.org/coi_disclosure.pdf.

J. Bachmann, N. Giese and M. Martignoni declare: no support from any organisation for the submitted work; no financial relationships with any organisations that might have an interest in the submitted work in the previous 3 years; no other relationships or activities that could appear to have influenced the submitted work.

## Authors’ contributions

Every author was involved in conception and design. AS carried out both molecular genetic studies and protein expression analysis. AS and JB drafted the manuscript. AS, JB, MM, HKR participated in analysis and interpretation of data.MM, NG, HKR revised it critically for important intellectual content and final approval of the version to be published. All authors read and approved the final manuscript.

## Pre-publication history

The pre-publication history for this paper can be accessed here:

http://www.biomedcentral.com/1471-2407/12/265/prepub

## Supplementary Material

Additional file 1 **Figure S1.**cDNA-AFLP: Comparison of 10 cachectic and 13 non-cachectic patients using one-primer-pair combination. Click here for file

Additional file 2 **Table S1.**Candidate biomarkers for cachexia progression. Legend: diff. - differential gene expression: ”+” cachexiaspecific upregulation,“-“cachexia-specific downregulation. Click here for file

Additional file 3 **Figure S2.**Full-length Western blotting of Egr-1. Click here for file
