# Supplementary figures and images for: Real-imaging cDNA-AFLP transcript profiling of pancreatic cancer patients: Egr-1 as a potential key regulator of muscle cachexia
Source: BMC Cancer. 2012 Jun 21;12:265. doi: 10.1186/1471-2407-12-265 (PMC3465185; doi:10.1186/1471-2407-12-265)

Figure S1

cDNA-AFLP Profiling of Muscle Cachexia

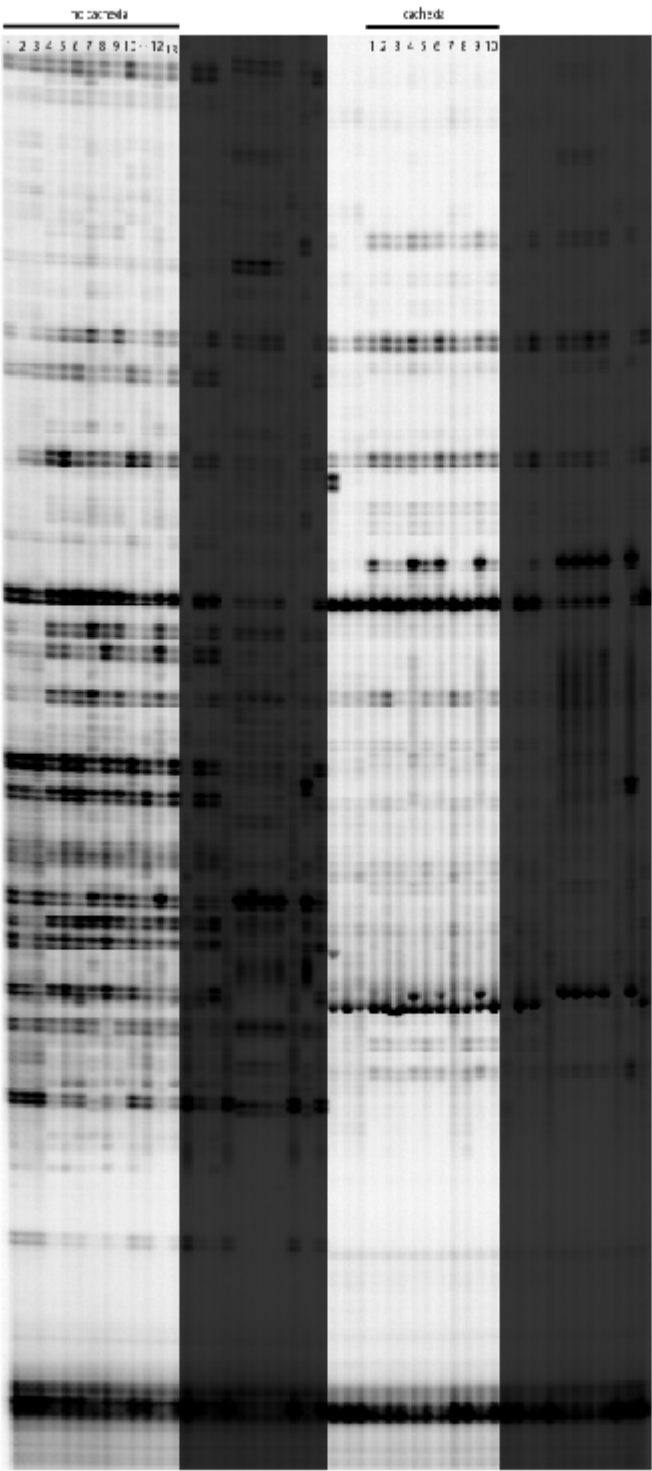

Supplement: Additional file 1 — Figure S1.cDNA-AFLP: Comparison of 10 cachectic and 13 non-cachectic patients using one-primer-pair combination. [file 1471-2407-12-265-S1.pdf]
