# Supplementary material for: Real-imaging cDNA-AFLP transcript profiling of pancreatic cancer patients: Egr-1 as a potential key regulator of muscle cachexia
Source: BMC Cancer. 2012 Jun 21;12:265. doi: 10.1186/1471-2407-12-265 (PMC3465185; doi:10.1186/1471-2407-12-265)
Supplement: Additional file 3 — Figure S2.Full-length Western blotting of Egr-1. [file 1471-2407-12-265-S3.pdf]

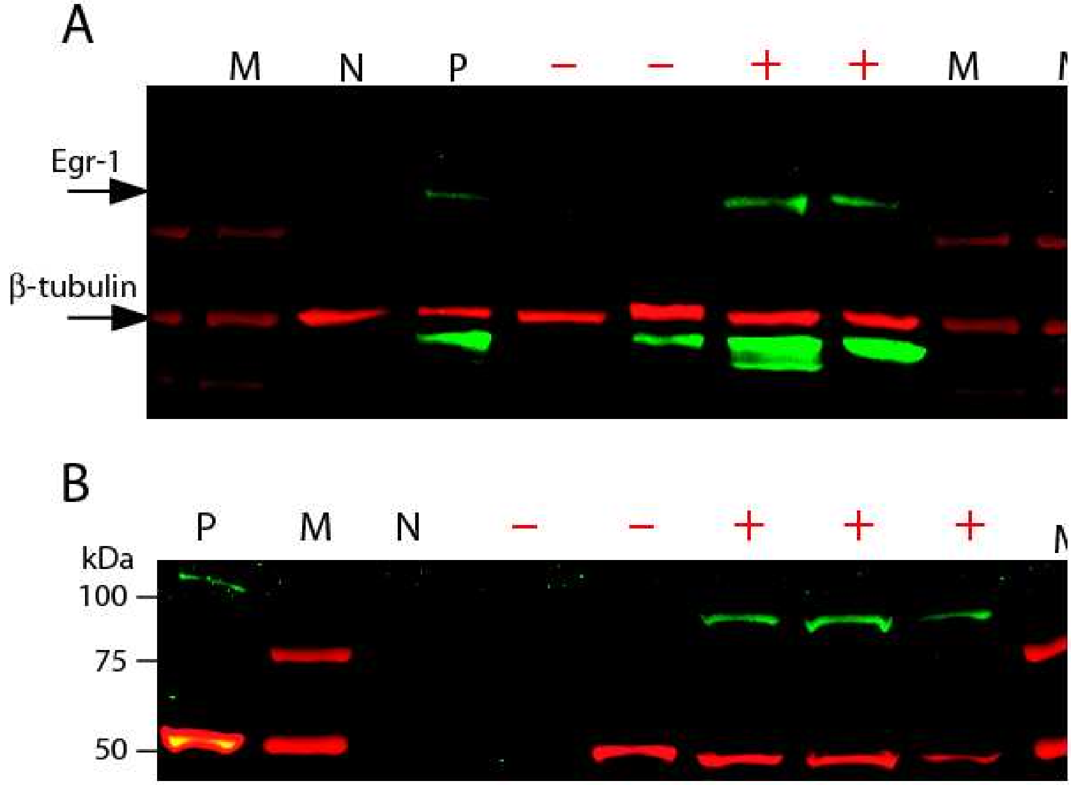

M - coomasie-blue prestained protein marker (Bio-RAD)
P - positive control (3T3 cell lysate, Santa Cruz)
N - negative control (293T cell lysate, Santa Cruz)
"-" - no cachexia (biopsy protein lysate)
"+" - cachexia (biopsy protein lysate)
